# Amyloid Plaque Reduction as a Surrogate Marker of Efficacy: Assessment of Amyloid PET and Change in CDR‐SB Utilizing Semi‐Mechanistic Model

**DOI:** 10.1002/alz.091955

**Published:** 2025-01-09

**Authors:** Brian A. Willis, Pratik Bhagunde, Robert Bell, Natasha Penner, Arnaud Charil, Michael C. Irizarry, Steven Hersch, Larisa Reyderman

**Affiliations:** ^1^ Eisai Inc., Nutley, NJ USA; ^2^ Deep Human Biology Learning (DHBL), Eisai Inc., Nutley, NJ USA

## Abstract

**Background:**

Lecanemab is a humanized IgG1 monoclonal antibody binding with high affinity to protofibrils of amyloid‐beta (Aβ) protein. In clinical studies, lecanemab has been shown to reduce markers of amyloid in early symptomatic Alzheimer’s disease (AD) and slow decline on clinical endpoints of cognition and function. Herein, a modeling approach was used to correlate amyloid reduction with change in rate of AD progression. This model was then utilized to evaluate amyloid PET as a surrogate marker of efficacy and to assess long‐term effect of lecanemab treatment on CDR‐SB.

**Method:**

Nonlinear mixed‐effects modeling assessed the correlation between amyloid PET and change in CDR‐SB. Data from the lecanemab phase 2 study (Study 201) and Clarity AD (Study 301) were pooled, and CDR‐SB scores were used with beta regression to fit a Richard’s function parameterized in terms of baseline CDR‐SB, intrinsic rate of disease progression, shape, and precision of the beta distribution. Inter‐subject variability was included on baseline CDR‐SB and intrinsic rate. Data from placebo‐treated subjects was used to establish a disease‐progression model; the effect of amyloid reduction on disease progression was defined using data from lecanemab‐treated subjects. Other baseline factors influencing disease progression were explored. Simulations were conducted to evaluate the impact of lecanemab treatment over 4 years.

**Result:**

Richard’s model well‐described AD progression. Baseline CDR‐SB was predicted by diagnosis and baseline mini‐mental state examination (MMSE) score. Intrinsic rate of disease progression was predicted by baseline amyloid PET and baseline MMSE score. Change in amyloid PET was a better predictor of drug effect than lecanemab exposure, demonstrating amyloid reduction as a surrogate marker of clinical efficacy. Simulations projected that the difference in CDR‐SB between lecanemab and placebo treated subjects continued increasing over the entire simulation period. Patients with low amyloid at baseline and less severe disease were projected to have slower disease progression than patients with higher amyloid or more severe disease at start of treatment.

**Conclusion:**

Amyloid reduction was shown to be predictive of slowing of AD progression. Individuals with less advanced disease and lower amyloid baseline were projected to have better outcomes with lecanemab treatment than more advanced patients.

